# Effect of Ultra-Fine Mineral Admixture on the Performance of Ultra-High-Performance Concrete

**DOI:** 10.3390/ma18040729

**Published:** 2025-02-07

**Authors:** Shiwei Bo, Li Gong, Haizhen Guo, Fucheng Guo, Tengfei Yao, Dingbang Wei

**Affiliations:** 1 School of Civil Engineering, Lanzhou Jiaotong University, Lanzhou 730070, China; 13240049@stu.lzjtu.edu.cn (S.B.); tfyao@lzjtu.edu.cn (T.Y.); 2 Gansu Transportation Planning, Survey and Design Institute Co., Ltd., Lanzhou 730030, China; 13993147030@163.com (H.G.); weidingbang@163.com (D.W.)

**Keywords:** ultra-high-performance concrete, ultrafine fly ash, ultrafine mineral powder, mechanical properties, pore structure, hydration degree

## Abstract

The ultra-high performance of ultra-high-performance concrete (UHPC) has led to a significant increase in cement mixing, resulting in an increase in CO_2_ emissions. Using mineral admixtures to replace cement partially is an effective way to reduce the cement amount. In this study, to investigate the effect of ultrafine fly ash (UFA) and ultrafine mineral powder (UMP) on the performance of UHPC, a series of experiments involving single admixture use of UFA, UMP, and their compound mixing were conducted. Results show that the cement dosage in UHPC can be reduced to 800 kg/m^3^ by mixing UFA and UMP. Compared with single mixing, compound mixing can effectively improve the fluidity and mechanical properties, and the optimal improvement effect is achieved when the dosage of UFA and UMP is equal. From the viewpoint of microscopic pore structure, the compound addition of ultrafine mineral admixtures can significantly improve the pore size distribution of UHPC by reducing the number of large pores and fissures and increasing the number of micropores and mesopores. From the perspective of microscopic hydration, ultrafine mineral admixtures can promote the cement hydration reaction, generating more C-S-H gels, enhancing the compactness of UHPC, and improving its mechanical properties and durability.

## 1. Introduction

Ultra-high-performance concrete (UHPC) is a kind of building material with silicate cement as the cementitious system and steel fiber as the reinforcing phase. Its compressive strength is more than 150 MPa. UHPC has good workability, mechanical properties, and durability. It is currently widely used as the most innovative and promising cementitious material [1,2,3,4]. UHPC has experienced more than 40 years of development, and nowadays, it is widely used in civil engineering. The first UHPC highway bridge, the Mars Hill Bridge, was built in the United States in 2006. The world’s first UHPC highway arch bridge, Wild Bridge, was built in Austria in 2010. The first UHPC bridge, the Luanbai Dry Canal Bridge in a railroad project, was built in China in 2006 [5]. Moreover, UHPC also has broad applications in water conservancy, electric power, and military fields [6,7,8,9].

To obtain ultra-high performance, UHPC has a significant increase in the amount of cement admixture compared to conventional concrete monoliths. The cement dosage of traditional C50 concrete is usually 500 kg/m^3^, which can be reduced to 375 kg/m^3^ by compounding mineral admixtures such as silica fume and fly ash [10], while the cement dosage of UHPC is usually more than 1000 kg/m^3^ [11], and the large amount of cement application implies a significant increase in CO_2_ emission. According to the data of the European Cement Association, the production of every ton of cement will produce at least half a ton of carbon dioxide [12]. The carbon dioxide emitted from the manufacture of cement has exceeded 7% of global carbon dioxide emissions [13], which are more than the emissions of all trucks in the world combined. Therefore, the development of a new ecologically friendly UHPC has become a goal pursued by many scholars.

Mineral admixtures have a good bead effect, filling effect, and reactive effect, and are inexpensive. It is an effective means to replace part of the cement by incorporating mineral admixtures in UHPC to reduce the amount of cement [14]. Through studying the design of the UHPC proportion based on the modified Andreasen–Andersen (MAA) model, Wen [15] found that when the mass ratio of cement, fly ash, and silica fume is 70:10:20, UHPC reaches the tightest stacking and more C-S-H gels can be generated, which can improve the microstructure of UHPC, and enhance the densification and compressive strength of the material. Wang [16] prepared self-dispersed particles by modifying silica fume and fly ash and found that they could effectively reduce UHPC viscosity and improve the working performance. Lee [17], by investigating the filling effect and gelling reaction of two different types of micro-silica in UHPC, found that in UHPC with a higher filling effect, C_3_S and C_2_S also undergo additional hydration at later ages, which increases the C-S-H gel ratio, decreases the porosity in the region with a pore size less than 10 nm, and significantly improves the UHPC compressive strength. Sun [18] found that the low carbon and low cost of UHPC can be achieved by partially replacing cement with both raw fly ash and silica fume. Research findings indicate that when raw fly ash replaced silica fume at an equal-mass ratio of 30%, the flexural strength of UHPC surged by 34%. Meanwhile, it also improves the fluidity of UHPC, reduces the total internal pore volume, and the matrix is more homogeneous and denser internally. In addition, many scholars have also studied the effect of compounding mineral admixtures such as fly ash and silica fume, fly ash and mineral powder, fly ash and blast furnace slag, etc., on the performance of UHPC. The results indicate that the compounding of mineral admixtures can not only reduce the cement dosage and be more ecologically friendly and cost-saving, but also effectively improve the mechanical properties, workability, durability, etc., and improve the pore structure, microstructure, etc., of UHPC, resulting in a better overall performance [19,20,21,22,23,24,25,26,27,28,29].

The above studies have shown that mineral admixtures, which are used for partial replacement of cement, can effectively improve various properties of UHPC and have become indispensable raw materials for its preparation. Currently, most of the scholars’ studies mainly focus on the effects of ordinary-sized mineral admixtures on the performance of UHPC, with limited research on the effects of ultrafine mineral admixtures. Ultrafine mineral admixtures refer to ultrafine powders with an average diameter of less than 10 μm, such as slag powder, fly ash, etc. These admixtures feature higher fineness, larger specific surface area, and stronger surface activity, enabling the more thorough exertion of the filling and activation effects of the admixtures. A few scholars have explored the idea that the partial substitution of ordinary fly ash with solely UFA can enhance the fluidity, mechanical characteristics, and micro-pore architecture of UHPC [30,31], and some others have examined the fact that the addition of nano-SiO_2_ and nano-CaCO_3_ can improve the mechanical performance and toughness of UHPC [32,33,34,35,36]. However, research on how the combined use of UFA and UMP impacts UHPC performance remains scarce. Therefore, experiments on the effect of single admixture of UFA, UMP, and compounding were carried out in this paper to investigate the effect of UFA and UMP on the performance of UHPC, which can provide a technical supplement for the preparation and application of low-carbon ecological UHPC.

To investigate the impact of UFA and UMP blending on the working performance, mechanical properties, pore structure, hydration products, and hydration degree in UHPC, comprehensive tests were conducted on collapse expansion, compressive strength, flexural tensile strength, pore structure analysis, hydration products, and degree of hydration in UHPC. The impact of varying proportions of two ultrafine mineral admixtures on the characteristics of UHPC was analyzed. This investigation can be used as a new modification method for UHPC to improve its various performances.

## 2. Materials and Methods

### 2.1. Raw Materials

**Figure 1:**
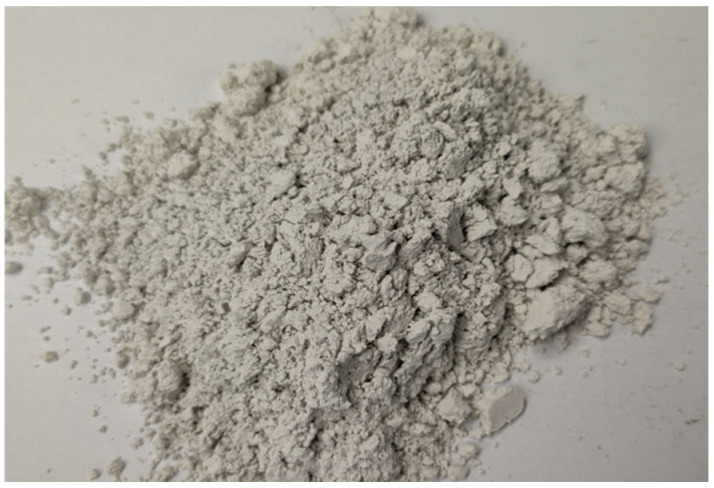
Ultrafine mineral powder appearance diagram.

**Figure 2:**
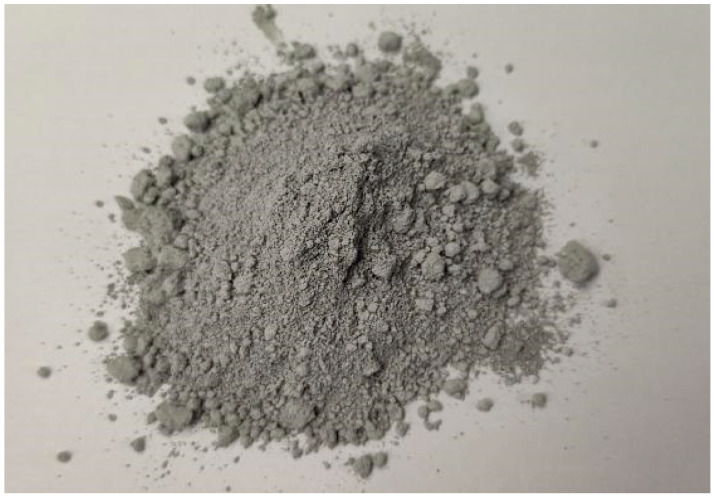
Ultrafine fly ash appearance diagram.

**Figure 3:**
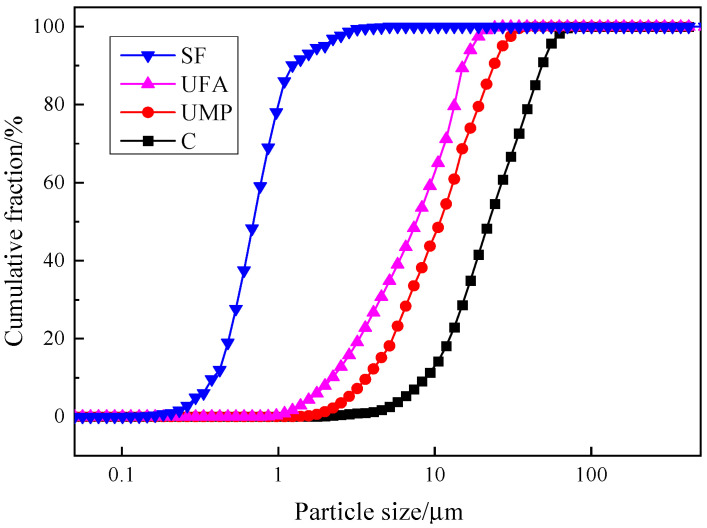
Particle size distribution of cementitious materials.

The cement adopted is ordinary Portland cement (C) of grade 52.5 from Gansu Qilianshan Cement Group Co., Ltd. in Lanzhou, China, and over 60% of the particles have a size of 10–30 μm. UMP, sourced from Ying’an Environmental Protection Material Technology Company located in Dezhou, China, as shown in Figure 1, has over 60% of particles sized 3–17 μm. UFA, sourced from Zhiqiang Fly Ash Plant located in Pingdingshan, China, as shown in Figure 2, has over 60% of particles sized 2–12 μm. Silica fume (SF), sourced from Lixinyuan Microsilica Powder Company located in Lanzhou, China, has over 60% of particles sized 0.4–0.9 μm. The particle size distributions of the above four materials are plotted into a curve as presented in Figure 3. Evidently, ranked by decreasing particle size, they are C > UMP > UFA > SF. SF, with the smallest particles, can effectively fill micropores. Cement (C), having the largest particles, is the primary material for hydration. The overall particle size of UFA is smaller than that of UMP, and both fall between SF and C.

**Figure 4:**
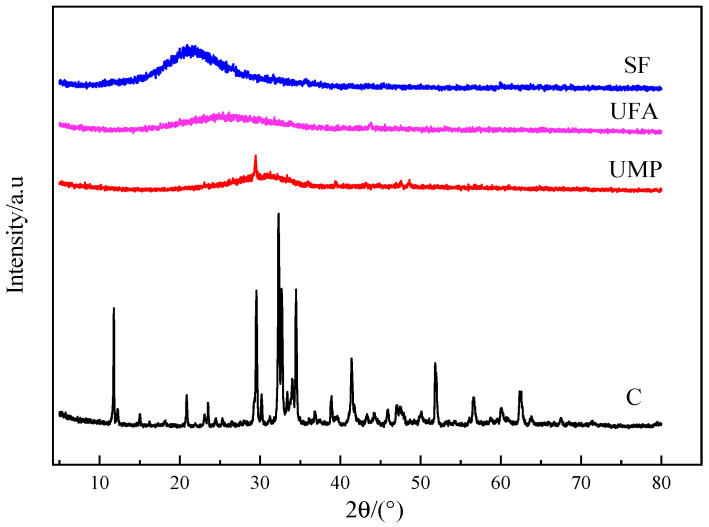
XRD spectra of cementitious materials.

The chemical composition of cement (C), ultrafine powder (UFA, UMP), and silica fume (SF) is shown in Table 1. The XRD spectra of the four gelling materials is shown in Figure 4. Compared to C, UMP, UFA, and SF have more silica and alumina, which can facilitate the formation of more minerals in UHPC, such as dicalcium silicate (C_2_S), tricalcium silicate (C_3_S), and tricalcium aluminate (C_3_A). These minerals are the main components influencing the strength of UHPC and can effectively enhance the mechanical and durability properties of UHPC [37].

Fine aggregate used is quartz sand with a particle size of 0.106~1.000 mm, sourced from Yongdeng Refined Quartz Sand Factory located in Lanzhou, China. Steel fiber (STF) is copper-plated steel fiber, sourced from Zhenqiang Fiber Company located in Shanghai, China. It has a diameter of 0.12 mm, a length of 13 mm, a tensile strength of more than 2850 MPa, and volume fraction of 2.8%. Dilatant (DLT) used is magnesium oxide-type dilatant, and the dosage of the mass fraction of the cementitious material is 2.42%. Defoamer (DFM) used is organosilicon defoamer, the dosage is 1.50% of the mass fraction of cementitious material. The water-reducing agent (HWRA) used is a powder high-performance polycarboxylic acid water-reducing agent sourced from Sobute New Materials Co., Ltd. located in Nanjing, China, and the water-reducing rate is more than 30%.

### 2.2. Sample Preparation

The cementitious materials (C, UFA, UMP, and SF) and admixtures (expansion agent, defoamer, powder water reducer) for the preparation of UHPC were first pre-mixed for 3 min to prevent agglomeration of ultrafine powder (UFA, UMP, and SF), and then the fine aggregate was added and mixed for 3 min to further ensure that the cementitious materials and the fine aggregates were uniformly dispersed, and then water was added and stirred for 2–3 min, and then after the slurry was completely fluidized, it was added with steel fiber and stirred for 3 min. The mixed UHPC was molded into specimens of 100 mm × 100 mm × 100 mm and 100 mm × 100 mm × 400 mm. Demolded after 1 day curing at room temperature. Subsequently, steam curing was carried out for 48 h under the conditions of a temperature of 90 °C and a relative humidity of 95%. Thereafter, the implementation of various experiments commenced.

The UHPC mixing ratio is shown in Table 2. The specimens used for XRD micro-composition and TG–DTG analysis were tested using net slurry, and the samples used for micropore structure analysis were tested using matrix slurry (without steel fibers).

### 2.3. Test Methods

**Figure 5:**
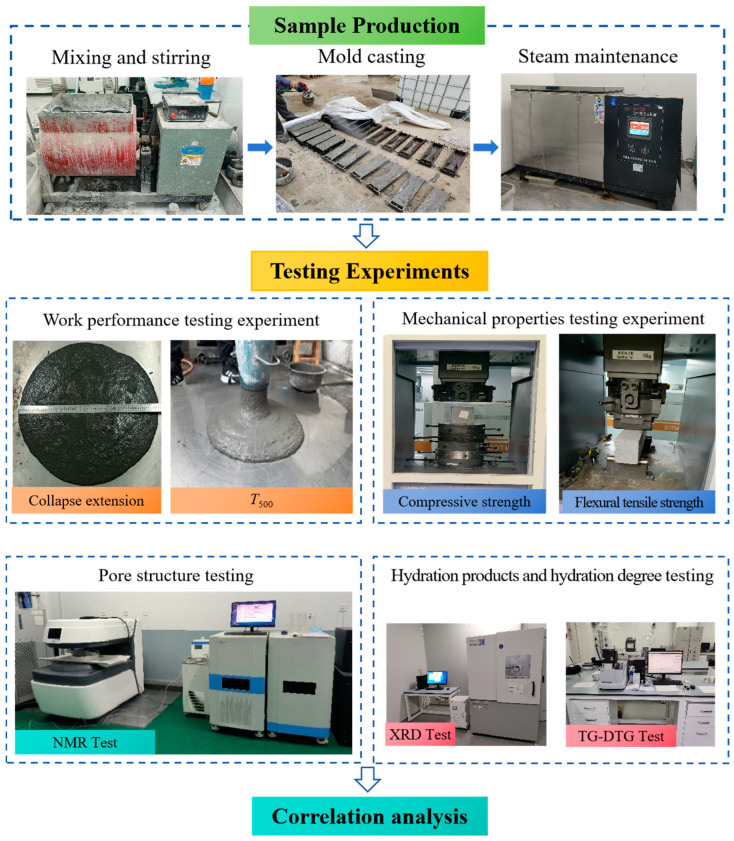
Experimental flowchart in this study.

To obtain the variation rules of working properties, mechanical properties, pore structure, hydration products, and hydration degree in UC-0~UC-100 samples, corresponding working properties, mechanical properties, and microscopic testing experiments should be carried out. The experimental process is shown in Figure 5. Specific requirements for each experiment are as follows:

(1) Work performance testing

The degree of collapse extension and the time required for the collapse extension to reach 500 mm are two important indexes to reflect the working performance of UHPC mixes. Therefore, to obtain the working performance of each sample, it is necessary to test their degree of collapse extension and *T*_500_ time. The test is carried out with the reference to the relevant requirements of *Standard for Test Method of Ultra-High Performance Concrete* (T/CECS 864-2021) [38].

(2) Mechanical properties testing

Compressive strength and flexural tensile strength are two key indexes to reflect the mechanical properties of the concrete. Therefore, to obtain the mechanical properties of each sample, it is necessary to test their compressive strength and flexural tensile strength. The test is carried out with reference to the relevant requirements of the *Standard for Test Methods of Concrete Physical and Mechanical Properties* (GB/T 50081-2019) [39].

(3) Pore structure testing

Pore structure is an important index to reflect the compactness of UHPC. By using the Macro MR12-150H-I low-field NMR microstructure analysis and imaging system from Niumag Analytical Instrument Corporation located in Suzhou, China, we can determine the number of micropores, mesopores, macropores, and fissures in each sample. And by calculating their percentages, we can intuitively grasp the compactness of UHPC.

(4) Hydration products and hydration degree testing

To reveal the influence of UFA and UMP on the microscopic properties of UHPC, it is necessary to test the XRD spectra of the hydration products of UHPC by using XRD equipment from Shimadzu Enterprise Management (Suzhou, China) Co., Ltd. And by analyzing the diffraction peak intensities of the hydration products, such as calcium hydroxide (CH), dicalcium silicate (C_2_S), tricalcium silicate (C_3_S), calix alumina (AFt), and calcium silicate hydrate (C-S-H), we can determine the ratio of UFA and UMP dosages for the highest C-S-H diffraction peak intensity. That is, the more C-S-H phase is generated, the more complete the UHPC hydration is.

## 3. Results and Discussion

### 3.1. Effect of Ultrafine Mineral Admixture on the Working and Mechanical Properties of UHPC

**Figure 6:**
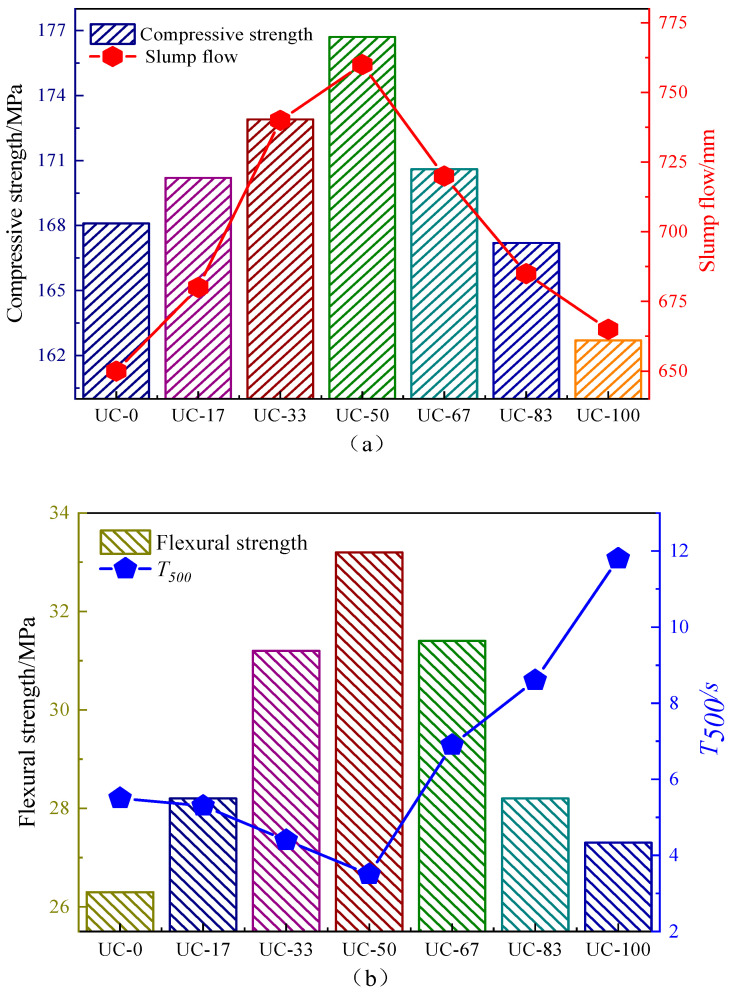
Effect of ultrafine mineral admixture on the working and mechanical properties of UHPC: (**a**) compressive strength and slump flow; (**b**) flexural strength and T_500_ time.

The UHPC collapse extensibility, *T*_500_ time, compressive strength, and flexural strength when different ratios of UMP replace UFA are shown in Figure 6.

From the viewpoint of workability, as can be seen from Figure 6a, with the increase in the ratio of UMP replacing UFA, the slump flow of UHPC shows a trend of first increasing and then decreasing. When the ratio of UMP replacing UFA is less than 50%, the slump flow value increases. The collapse extension degree of UC-0, UC-17, UC-33, and UC-50 is 650 mm, 680 mm, 740 mm, and 760 mm, respectively. Using UC-0 as the control group, the collapse extensibility of UC-17, UC-33, and UC-50 is improved by 4.6%, 13.8%, and 16.9%. Conversely, when the ratio of UMP replacing UFA is more than 50%, the slump flow value decreases. The collapse extension degree of UC-50, UC-67, UC-83, and UC-100 is 760 mm, 720 mm, 685 mm, and 665 mm. Using UC-50 as the control group, the collapse extensibility of UC-67, UC-83, and UC-100 decreases by 5.3%, 9.9%, and 12.5%, respectively.

As shown in Figure 6b, with the increase in the ratio of UMP replacing UFA, the *T*_500_ time of UHPC shows a trend of first decreasing and then increasing. When the ratio of UMP replacing UFA is less than 50%, *T*_500_ time decreases. The *T*_500_ time of UC-0, UC-17, UC-33, and UC-50 is 5.5 s, 5.3 s, 4.4 s, and 3.5 s, respectively. Using UC-0 as the control group, the *T*_500_ time of UC-17, UC-33, and UC-50 is reduced by 3.6%, 20.0%, and 36.4%. Conversely, when the ratio of UMP replacing UFA is more than 50%, *T*_500_ time increases. The T_500_ time of UC-50, UC-67, UC-83, and UC-100 is 3.5 s, 6.9 s, 8.6 s, and 11.8 s in order. Using UC-50 as the control group, the *T*_500_ time of UC-67, UC-83, and UC-100 increases by 97.1%, 145.7%, and 237.1%, respectively.

Based on the above analysis, it can be seen that, compared with UC-0 and UC-100 single doping of UFA and UMP, respectively, UC-17~UC-83 compound doping of UFA and UMP can significantly improve the degree of expansion of UHPC collapse and *T_500_* time, which indicates that the effect of complex doping on the working performance of UHPC is significantly better than that of single doping. When the ratio of UMP replacing UFA is equal to 50%, the work performance is the best. The main reason is that, as shown in Figure 3, the particle sizes of UFA and UMP are smaller than that of cement particles, which can fill the gaps between the UHPC particles more fully and release the free water needed to fill these gaps, thus improving the fluidity of UHPC.

From the viewpoint of mechanical properties, from Figure 6a, with the increase in the ratio of UMP replacing UFA, the compressive strength of UHPC shows a trend of first increasing and then decreasing. When the ratio of UMP replacing UFA is less than 50%, the compressive strength of UHPC gradually grows. The compressive strengths of UC-0, UC-17, UC-33, and UC-50 are 168.1 MPa, 170.2 MPa, 172.9 MPa, and 176.7 MPa in succession. Using UC-0 as the control group, the UC-17, UC-33, and UC-50 compressive strengths are increased by 1.3%, 2.8%, and 5.1%, respectively. When the ratio of UMP replacing UFA is more than 50%, with the further increase in UMP dosage, the compressive strength of UHPC shows a gradual decrease, and the compressive strengths of UC-50, UC-67, UC-83, and UC-100 are 176.7 MPa, 170.6 MPa, 167.2 MPa, and 162.7 MPa in order. Using UC-50 as the control group, the compressive strengths of UC-67, UC-83, and UC-100 decrease by 3.5%, 5.4%, and 7.9%, respectively. It can be seen from the above analysis that the combined addition of UFA and UMP can significantly improve the compressive strength of UHPC, where the compressive strength reaches its maximum when the dosages of UFA and UMP are equal. This is mainly because the particle size distribution area of UFA and UMP particles is between cement and silica fume, which can play a better filling role, and a large amount of SiO_2_ and Al_2_O_3_ in UFA and UMP can react with calcium hydroxide (CH) to generate more hydrated calcium silicate (C-S-H) gels during cement hydration, which makes the microstructure of UHPC denser and, thus, improves the compressive strengths of UHPC [40].

From Figure 6b, with the increase in the ratio of UMP replacing UFA, the flexural tensile strength of UHPC also shows a trend of first increasing and then decreasing. When the ratio of UMP replacing UFA is less than 50%, the flexural tensile strengths of UC-0, UC-17, UC-33, and UC-50 are 26.3 MPa, 28.2 MPa, 31.2 MPa, and 33.2 MPa in order. Using UC-0 as the control group, the UC-17, UC-33, and UC-50 flexural tensile strengths are improved by 7.2%, 18.6%, and 26.2%, respectively. When the ratio of UMP replacing UFA is more than 50%, the flexural tensile strength of UHPC shows a gradual decrease with the further increase in UMP dosage. The flexural tensile strengths of UC-50, UC-67, UC-83, and UC-100 are 33.2 MPa, 31.4 MPa, 28.2 MPa, and 27.3 MPa in order. Using UC-50 as the control group, UC-67, UC-83 and UC-100 flexural tensile strengths are reduced by 5.4%, 15.1% and 17.8%, respectively. It can be known from the above analysis that the combined addition of UFA and UMP can also improve the flexural strength of UHPA, where the most optimal improvement in flexural strength is achieved when the admixture dosages of UFA and UMP are equivalent. The main reason for the increase in the flexural tensile strength of UHPC is attributed to the filling and active effects of UFA and UMP, which optimize the transition zone between the interface of the UHPC matrix and the steel fibers, and enhance the anchoring effect on the steel fibers, and, therefore, significantly improve the flexural tensile performance of UHPC [41].

### 3.2. Analysis of the Effect of Ultrafine Mineral Admixture on the Pore Structure of UHPC

**Figure 7:**
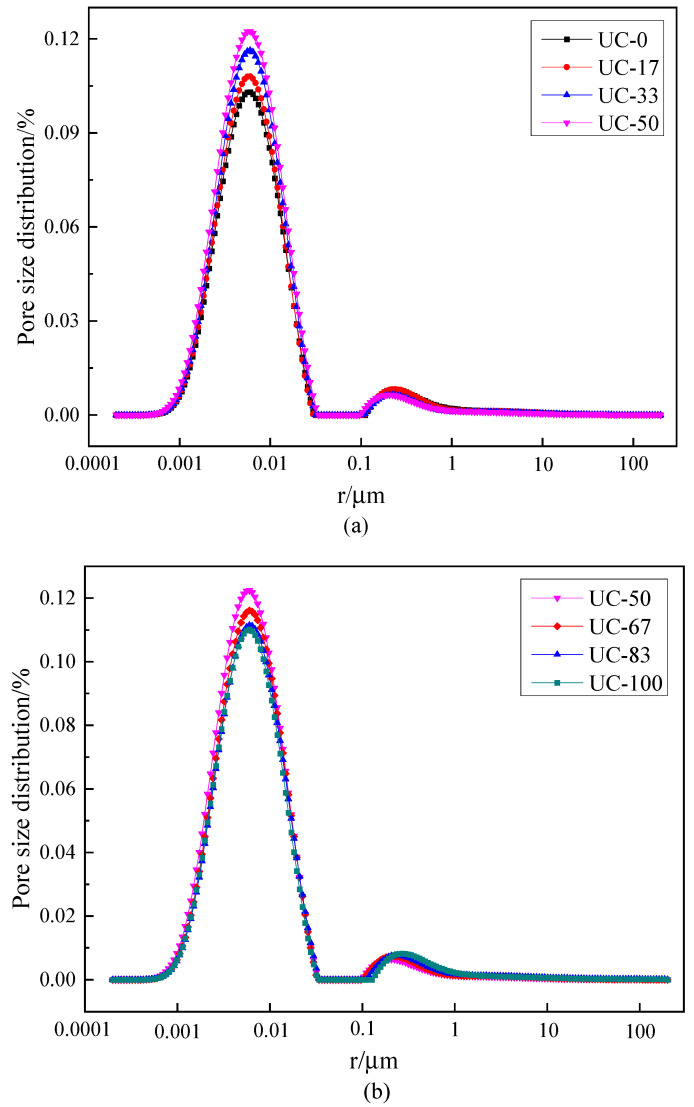
Pore size distribution of UHPC when UMP and UFA were mixed: (**a**) ratio of UMP replacing UFA is less than 50%; (**b**) ratio of UMP replacing UFA is larger than 50%.

To further investigate the influence law of UFA and UMP on the pore structure of UHPC, the internal pores of the specimens are classified into four types of intervals according to pore size distribution, namely, micropore (r ≤ 0.01 μm), mesopore (0.01 μm < r < 0.05 μm), macropore (0.05 μm ≤ r < 1 μm), and fissure (r ≥ 1 μm) in accordance with the relevant research results [42]. The pore size distribution results of UHPC when UMP and UFA were mixed are shown in Figure 7. The internal pore distribution of the specimen is shown in Table 3.

As can be seen from Figure 7a and Table 3, the UHPC pore size distribution curve shifts slightly to the left with the increase in UMP dosage when the ratio of UMP replacing UFA is less than 50%. The peak area of the pore size distribution of the small pores increases slightly. This indicates that the use of UMP replacing UFA for compounding can effectively improve the pore size distribution of UHPC. The numbers of micropores and mesopores are increased, and the numbers of macropores and fissures are decreased. The pore size percentage distribution of UC-0, UC-17, UC-33, and UC-50 is shown in Table 3. Using UC-0 as the control group, the microporous percentage of UC-17, UC-33, and UC-50 increases by 0.94%, 1.28%, and 1.91%, while the mesoporous percentage increases by −0.44%, 1.16%, and 0.29%. The macroporous percentage decreases by 0.31%, 1.78%, and 1.82%, while the fissure percentage decreases by 0.19%, 0.65%, and 0.65%.

As can be seen from Figure 7b and Table 3, the pore structure of UHPC deteriorates to different degrees with the further increase in UMP dosage. Using UC-50 as the control group, the pore size distribution curves of UC-67, UC-83, and UC-100 shift slightly to the right. The numbers of micropores and mesopores are decreased, and the numbers of macropores and fissures are increased. The micropores proportions of UC-67, UC-83, and UC-100 are decreased by 0.04%, 1.04%, and 1.7%, while the mesopores proportions are decreased by 0.59%, 0.16%, and −0.02%. The macropores proportions are increased by 0.71%, 1.07%, and 1.46%, while the fissures increases by 0.19%, 0.41%, and 0.51%.

As can be seen from Table 3, UHPC pores mainly consist of micropores, accounting for 72.72% to 74.63% of the total pore volume. The following is mesoporous, accounting for 19.86 to 21.46% of the total pore volume. The microporous in UHPC pores accounts for 3.49% to 5.31% of the total pore volume. The smallest volume in UHPC pores is the fissure, with a share of 1.01% to 1.66%.

### 3.3. Analysis of the Effect of Ultrafine Mineral Admixture on UHPC Hydration Products

**Figure 8:**
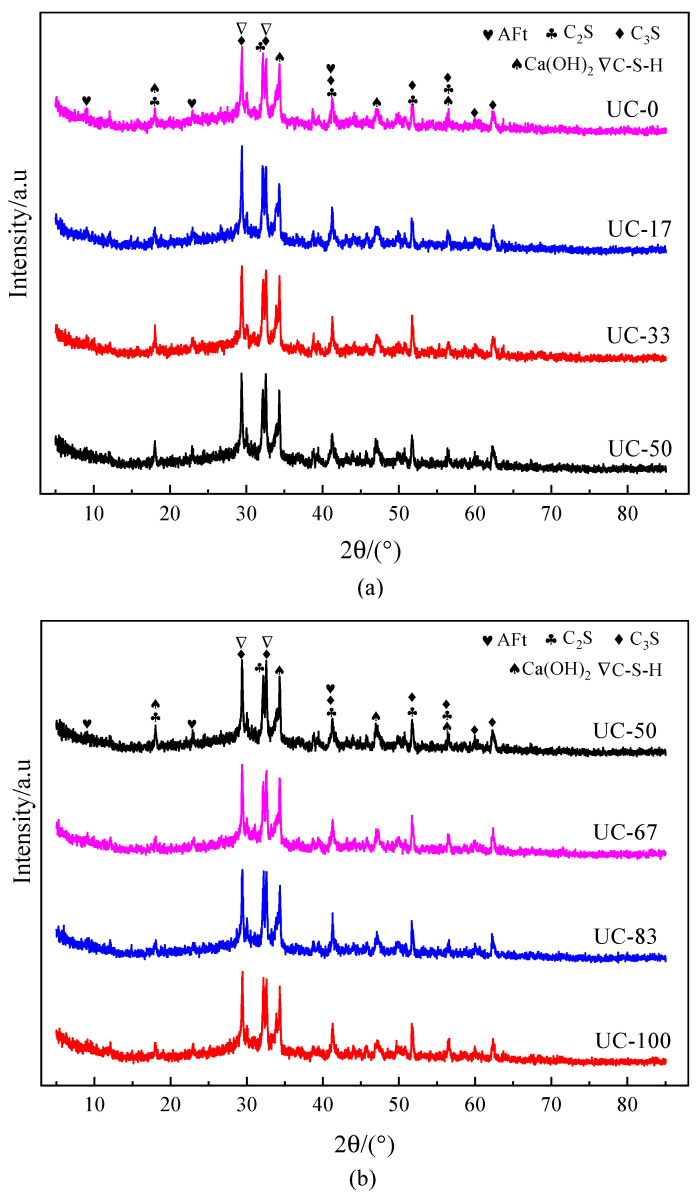
XRD spectra of UHPC when UMP and UFA were added: (**a**) ratio of UMP replacing UFA is less than 50%; (**b**) ratio of UMP replacing UFA is larger than 50%.

The XRD spectra of UHPC net slurry hydration products when UMP and UFA were added are shown in Figure 8, where Figure 8a shows when the ratio of UMP replacing UFA is less than 50%, while Figure 8b shows when the ratio of UMP replacing UFA is more than 50%.

As can be seen from Figure 8a, the types of XRD diffraction peaks of the net slurry after the completion of UHPC steam curing are similar, but the intensity of the diffraction peaks of the individual phases is different. Significant diffraction peaks of hydration products such as calcium hydroxide (CH), dicalcium silicate (C_2_S), tricalcium silicate (C_3_S), ettringite (AFt), and hydrated calcium silicate (C-S-H), etc., appear in different samples, and the hydration reaction of C_2_S and C_3_S mineral phases occurs during hydration to produce hydration products such as C-S-H and AFt, etc. A comparative analysis of the diffraction peaks of hydration products under different dosages of UMP replacing UFA shows that with the increase in UMP dosage, the CH diffraction peaks of UC-0, UC-17, UC-33, and UC-50 groups increase significantly at (2θ = 18°), which indicates that more CH products are produced. The C_2_S diffraction peaks at (2θ = 32°) decrease gradually, and the C-S-H diffraction peaks increase significantly. This is mainly because the incorporation of ultrafine admixture can promote the system to undergo secondary hydration reactions and produce more C-S-H phases [43], which is conducive to the promotion of specimen strength development.

As can be seen from Figure 8b, the XRD diffraction peaks of the net slurry after the completion of UHPC steam curing are also the hydration products such as CH, C_2_S, C_3_S, Aft, and C-S-H, etc. With the further increase in UMP dosage, the CH diffraction peaks of the UC-50, UC-67, UC-83, and UC-100 groups are significantly reduced at (2θ = 18°), indicating that the amount of CH produced starts to decrease. The C_2_S diffraction peak (2θ = 32°) gradually increases, and the C-S-H diffraction peak obviously decreases, which proves that when the ratio of UMP replacing UFA is equal to 50%, the secondary hydration reaction in the system occurs most completely and the largest amount of hydration products are generated.

### 3.4. Analysis of the Effect of Ultrafine Mineral Admixture on the Degree of Hydration of UHPC

**Figure 9:**
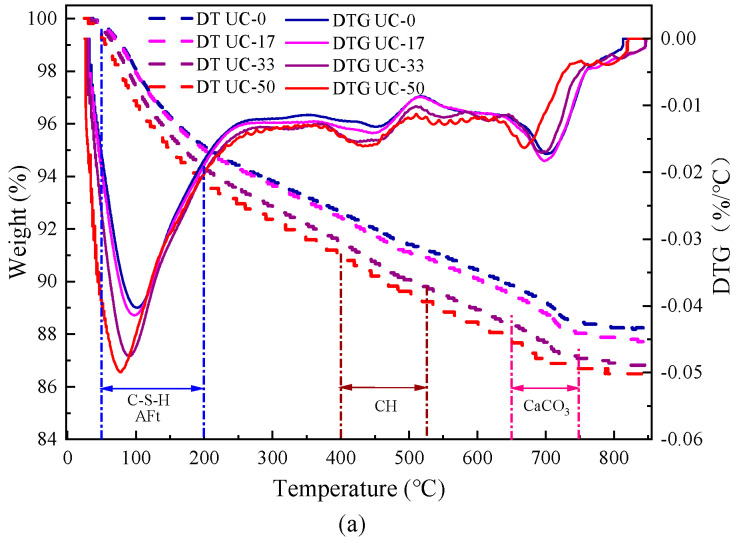

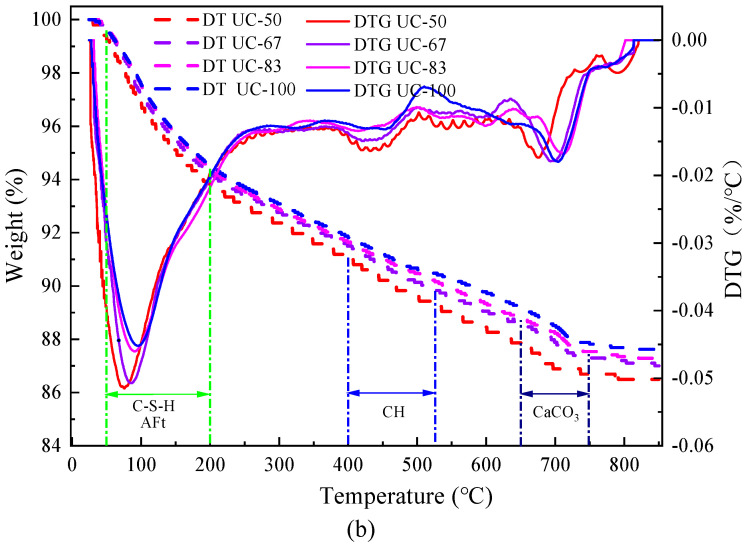
TG–DTG curves of UHPC when UMP and UFA were added: (**a**) ratio of UMP replacing UFA is less than 50%; (**b**) ratio of UMP replacing UFA is larger than 50%.

The TG–DTG curves of UHPC when UMP and UFA were added are shown in Figure 9, where Figure 9a shows when the ratio of UMP replacing UFA is less than 50%, and Figure 9b shows when the ratio of UMP replacing UFA is more than 50%.

From Figure 9a, the first obvious heat absorption peak in the DTG curves occurs between 60 °C and 200 °C, which is mainly a result of the dehydration of hydrated calcium silicate gel (C-S-H) and ettringite (AFt), exhibiting a significant enhancement with the increase in the dosage of UMP replacing UFA. The second obvious heat absorption peak occurs between 370 °C and 530 °C, which is mainly due to the dehydration decomposition of CH and shows an obvious increasing trend with the increase in UMP dosage. The third distinct heat absorption peak is located between 660 °C and 770 °C and corresponds to the dehydration decomposition of calcium carbonate (CaCO_3_). Based on the DTG curves, the common weight loss of C-S-H and AFt between 60 °C and 200 °C is calculated, and the weight loss of UC-17, UC-33, and UC-50 increases by 0.07%, 0.85%, and 1.43%, respectively, using UC-0 as the control group. The weight loss of CH between 370 °C and 530 °C is calculated, and the weight loss of UC-17, UC-33, and UC-50 increases by 0.21%, 0.34%, and 0.38%, respectively, using UC-0 as the control group. This indicates that when using UMP to replace UFA for compound admixture, the CH consumption rises with the increase in UMP replacing UFA, and more C-S-H gels are generated.

From Figure 9b, the first obvious heat absorption peak in the DTG curves occurs between 50 °C and 200 °C, mainly as a result of the dehydration of hydrated calcium silicate gel (C-S-H) and ettringite (AFt), which begins to exhibit a significant decrease with the increase in UMP dosage. The second obvious heat absorption peak mainly occurs between 400 °C and 520 °C, which is mainly due to the dehydration decomposition of CH, and also shows a significantly lower trend with the increase in UMP dosage. The third distinct heat absorption peak is located between 650 °C and 750 °C and corresponds to the dehydration decomposition of calcium carbonate (CaCO_3_). Based on the DTG curves, the common weight loss of C-S-H and AFt between 50 °C and 200 °C is calculated, and the weight loss of UC-67, UC-83, and UC-100 is reduced by 0.53%, 0.69%, and 0.86%, respectively, using UC-50 as the control group. The weight loss of CH between 400 °C and 520 °C is calculated and the weight loss for UC-67, UC-83, and UC-100 is reduced by 0.19%, 0.33%, and 0.49%, respectively, using UC-50 as the control group. This indicates that when the ratio of UMP replacing UFA is more than 50%, with the continuous increase in UMP added, the consumption of CH gradually decreases, and the quantity of the generated C-S-H gel also diminishes progressively.

## 4. Conclusions

In this study, to investigate the influence of UFA and UMP compound mixing on the performance of UHPC, the different proportions of the two kinds of mineral admixture were admixed into UHPC. The working performance, mechanical properties, pore structure, hydration products, and degree of hydration after the admixture were detected and summarized through experiments, and the following conclusions can be obtained:

(1) The cement dosage in UHPC can be chosen as 800 kg/m^3^ by mixing mineral admixtures UFA and UMP. Compared with single mixing of UFA or UMP, compound mixing can improve the working and mechanical properties of UHPC more effectively, and the degree of collapse extension can be improved by 4.6~16.9%, *T*_500_ time can be reduced by 3.6~36.4%, the compressive strength can be improved by 1.3~7.9%, and the flexural tensile strength can be improved by 5.4~26.2%, and the improvement effect is optimal when the admixture proportion of UFA and UMP is equal, i.e., both of them are 150 kg/m^3^.
(2) From the perspective of microscopic pore structure, ultrafine mineral admixture compounding can significantly improve the pore size distribution of UHPC, reduce the number of macropores and fissures, with a maximum reduction of 1.82% and 0.65%, and increase the number of micropores and mesopores, with a maximum increase of 1.91% and 1.16%, and the improvement effect is optimal when the admixtures of UFA and UMP are equal.
(3) From the perspective of microscopic hydration products, the XDR diffraction peaks of the hydration products, such as CH and C-S-H, etc., measured when the ultrafine mineral admixture is compounded, increase significantly, which promotes the secondary hydration reaction of the UHPC system, generates more C-S-H gels, and improves the compactness of the UHPC.
(4) From the perspective of microscopic hydration degree, ultrafine mineral admixture compounding can promote the increase in CH consumption, higher hydration degree, generation of more C-S-H gels, and denser UHPC, ensuring corresponding better mechanical properties and durability performance.

## Figures and Tables

**Table 1:** Chemical composition of cementitious materials (mass fraction, %).

Cementitious Materials	CaO	SiO_2_	Al_2_O_3_	SO_3_	Fe_2_O_3_	MgO	K_2_O	Na_2_O	TiO_2_	MnO	ZnO	P_2_O_5_
C	60.79	21.80	6.95	3.78	3.25	1.93	0.50	0.32	0.20	0.17	0.10	—
UFA	6.91	51.20	21.23	1.89	8.39	0.84	2.60	2.62	1.20	—	1.68	0.85
UMP	34.31	32.08	20.30	2.45	0.34	8.15	0.40	0.61	0.82	0.21	—	—
SF	0.66	91.3	0.72	0.38	0.53	1.29	1.35	2.33	—	—	0.02	—

**Table 2:** Mix ratio of UHPC.

Sample	Amount of Material/(kg/m^3^)										
	Water	C	UFA	UMP	Ratio of UMP Replacing UFA	SF	Sand	STF	HWRA	DFM	DLT
UC-0	195	800	300	0	0%	120	850	220	10	2	20
UC-17	195	800	250	50	17%	120	850	220	10	2	20
UC-33	195	800	200	100	33%	120	850	220	10	2	20
UC-50	195	800	150	150	50%	120	850	220	10	2	20
UC-67	195	800	100	200	67%	120	850	220	10	2	20
UC-83	195	800	50	250	83%	120	850	220	10	2	20
UC-100	195	800	0	300	100%	120	850	220	10	2	20

**Table 3:** Changes in pore structure of UHPC with different ultrafine mineral admixtures.

Sample	Micropore/%	Mesopore/%	Macropore/%	Fracture/%
UC-0	72.72	20.30	5.31	1.66
UC-17	73.66	19.86	5.00	1.47
UC-33	74.00	21.46	3.53	1.01
UC-50	74.63	20.59	3.49	1.01
UC-67	74.59	20.00	4.20	1.20
UC-83	73.59	20.43	4.56	1.42
UC-100	72.93	20.61	4.95	1.52

## Data Availability

The original contributions presented in the study are included in the article, further inquiries can be directed to the corresponding author.

## References

[1] Zhang Y., Zhang W., Chen Z. (2017). Summary of Ultra High Performance Concrete: Design and Preparation, Microstructure, Mechanics and Durability, and Engineering Applications. Mater. Rep..

[2] Shi C., Wu Z., Xiao J. (2015). A review on ultra high performance concrete: Part I. Raw materials and mixture design. Constr. Build. Mater..

[3] Wang D., Shi C., Wu Z. (2015). A review on ultra high performance concrete: Part II. Hydration, microstructure and properties. Constr. Build. Mater..

[4] Ullah R., Qiang Y., Ahmad J., Vatin N.I., El-Shorbagy M.A. (2022). Ultra-High-Performance Concrete (UHPC): A State-of-the-Art Review. Materials.

[5] Shao X., Qiu M., Yan B., Luo J. (2017). A Review on the Research and Application of Ultra-high Performance Concrete in Bridge Engineering Around the World. Mater. Rep..

[6] Teng J., Xiang Y., Yu T., Fang Z. (2019). Development and mechanical behaviour of ultra-high-performance seawater sea-sand concrete. Adv. Struct. Eng..

[7] Lai V., Hejazi F., Saleem S. (2020). The construction process for pre-stressed ultra high performance concrete communication tower. PLoS ONE.

[8] Ning H., Ren H., Wang W., Nie X. (2023). Impact Resistance of Ultra-High-Performance Concrete Composite Structures. Materials.

[9] Muhammad U., Shamsad A., Akhtar A., Husain J. (2020). Shielding performance of heavy-weight ultra-high-performance concrete against nuclear radiation. Prog. Nucl. Energy.

[10] Chen Z., Jiang H., Wang P. (2024). Optimization of mineral admixture ratio in C50 concrete. Highway.

[11] Yu R., Spiesz P., Brouwers H. (2014). Mix design and properties assessment of Ultra-High Performance Fibre Reinforced Concrete (UHPFRC). Cem. Concr. Res..

[12] CEMBUREAU Cementing the European Green Deal-Reaching Climate Neutrality Along the Cement and Concrete Value Chain by 2050. https://cembureau.eu/media/kuxd32gi/cembureau-2050-roadmap_final-version_web.pdf.

[13] IEA (2018). Cement Technology Roadmap Plots Path to Cutting CO_2_ Emissions.

[14] Randl N., Steiner T., Ofner S., Baumgartner E. (2014). Development of UHPC mixtures from an ecological point of view. Constr. Build. Mater..

[15] Wen D., Wei D., Wu L. (2022). Design and characteristic analysis of UHPC matrix mix proportion based on MAA model. J. Build. Mater..

[16] Wang D., Zhang S., Yao S. (2021). The influence of modified silica fume and fly ash on the performance of ultra-high performance concrete (UHPC). China Concr. Cem. Prod..

[17] Lee N., Koh K., Kim M., Ryu G. (2018). Uncovering the role of micro silica in hydration of ultra-high performance concrete (UHPC). Cem. Concr. Res..

[18] Sun J., Wang H., Lan J., Zhou K. (2022). Effect of Raw Fly Ash on Performance of Ultra-High Performance Concrete. Bull. Chin. Ceram. Soc..

[19] Wu Z., Shi C., He W. (2017). Comparative study on flexural properties of ultra-high performance concrete with supplementary cementitious materials under different curing regimes. Constr. Build. Mater..

[20] Wang B., Li X., Xu F., Su H. (2012). The effects of mineral admixtures on the basic properties of C100~C120 ultra-high strength concrete. China Concr. Cem. Prod..

[21] Ahmed M., Gamal M., Mohamed A. (2021). Durability and microstructure of eco-efficient ultra-high-performance concrete. Constr. Build. Mater..

[22] Ganesh P., Murthy A. (2019). Tensile behaviour and durability aspects of sustainable ultra-high performance concrete incorporated with GGBS as cementitious material. Constr. Build. Mater..

[23] Ding D., Guo Z., Zhang W., Zhang X. (2024). Effects of Fly Ash and Steel Fiber on Abrasion Resistance of Ultra-High Performance Concrete. Bull. Chin. Ceram. Soc..

[24] Qin Z., Zhou Z., Zhu G. (2023). Effect of fly ash-mineral powder ultrafine composite mineral admixture on durability of concrete. New Build. Mater..

[25] Xing C., Deng Y., Da J., Li M. (2022). Research on the influence of silica fume-fly ash composite mineral admixture on concrete performance. New Build. Mater..

[26] Ge L., Zhang Y., Feng Z., Li H. (2023). Performance and microscopic study of various mineral admixtures on reactive powder concrete. Mater. Lett..

[27] Fu D., Xia C., Xu S., Zhang C. (2022). Effect of concrete composition on drying shrinkage behavior of ultra-high performance concrete. J. Build. Eng..

[28] Guan D., Pan T., Guo R., Wei Y., Qi R., Fu C., Zhang Z., Zhu Y. (2024). Fractal and Multifractal Analysis of Microscopic Pore Structure of UHPC Matrix Modified with Nano Silica. Fractal Fract..

[29] Lin J., Zhang Y., Guo Z., Du H. (2024). Impact of synthetic fibers on spalling and intrinsic pore structure of ultra-high performance concrete (UHPC) under elevated temperatures. Constr. Build. Mater..

[30] Cao R., Zhou M., Zou Q., He Y. (2019). The effect of ultra-fine fly ash on the rheological, mechanical properties, and microstructure of ultra-high performance concrete. Mater. Rep..

[31] He X., Chen Y., Zhang X., Zhu H., Li H. (2024). Effect of Ultrafine Fly Ash Morphology on Ultra-high Performance Concrete (UHPC) Properties and Structure. Mater. Rep..

[32] Huang Z., Cao F. (2012). Effects of Nano-materials on the Performance of UHPC. Mater. Rep..

[33] Rong Z., Jiang G., Sun W. (2015). Effects of nano-SiO_2_ and nano-CaCO_3_ on properties of ultra-high performance cementitious composites. J. Southeast Univ. Nat. Sci. Ed..

[34] Huang K., Xie J., Wang R., Feng Y., Rao R. (2021). Effects of the combined usage of nanomaterials and steel fibres on the workability, compressive strength, and microstructure of ultra-high performance concrete. Nanotechnol. Rev..

[35] Liu C., He X., Deng X., Wu Y., Zheng Z. (2020). Application of nanomaterials in ultra-high performance concrete: A review. Nanotechnol. Rev..

[36] Yoo D., Oh T., Banthia N. (2022). Nanomaterials in ultra-high-performance concrete (UHPC)—A review. Cem. Concr. Comp..

[37] Wang F., Chen P., Li X., Zhu B. (2018). Effect of Colloidal Silica on the Hydration Behavior of Calcium Aluminate Cement. Materials.

[38] (2021). Standard for Test Method of Ultra-High Performance Concrete.

[39] (2019). Standard for Test Methods of Concrete Physical and Mechanical Properties.

[40] Felipe B., Kwesi S., Duan W. (2022). A century of research on calcium silicate hydrate (C–S–H): Leaping from structural characterization to nanoengineering. J. Am. Ceram. Soc..

[41] Wei X., Zhu H., Chen Q., Ju J. (2023). Microstructure-based prediction of UHPC’s tensile behavior considering the effects of interface bonding, matrix spalling and fiber distribution. Cem. Concr. Comp..

[42] Deng X., Gao X., Wang R., Zhao C. (2021). Study on Frost Resistance and Pore Distribution Change of Recycled Concrete. Mater. Rep..

[43] Wang X., Lee H. (2010). Modeling the hydration of concrete incorporating fly ash or slag. Cem. Concr. Res..

